# The Story of the Insane from Year to Year

**Published:** 1890-10-18

**Authors:** 


### The Story of the Insane from Year to Year.

[Annual Reports, Pamphlets, &c., should be addressed to The Editor, The Lodge, Porchester Square, London, W.]

### NORTH RIDING ASYLUM, YORK.

On the last day of 1889 this asylum contained 659 patients, showing an increase of 37 on the year previous. The admissions, discharges, and deaths stand respectively at 167, 74, and 57. Among the admissions were 14 general paralytics, 8 epileptics, and 32 dements. It is, therefore, not surprising to find that the recovery rate was low, namely, 28·7 per cent. of the admissions. If those London County Councillors and others who are crying out for an acute hospital for the insane were to study the reports of our county asylums there would probably be less nonsense talked and written. The asylum was visited by measles, and as the wards were full at the time some difficulty was experienced in isolating the cases. A new hospital for sixteen patients is to be built at an estimated expense of £4,500. It ought to be an extremely good one; indeed, it is difficult to see how so much could be expended on a building intended to accommodate only sixteen cases. The committee of visitors say that "they have satisfied themselves that the patients are well cared for, and that their food is good and sufficient, and that the building is preserved in a clean and efficient condition." The weekly rate of maintenance is 9s. 4d. for paupers. Private patients pay from 14s. to 31s. 6d. a-week. The Commissioners say that the wards "are bright and comfortable, and the general arrangements therein for the care and treatment of the inmates deserve favourable notice."

### SALOP AND MONTGOMERY ASYLUM.

The annual report shows that 695 patients were in residence at the close of the year, and this number included 13 private patients. The admissions were 175, the discharges 53, and the deaths 80. Calculated in the usual way the recoveries stand at 30·28, and the deaths at 11·47. The weekly cost of maintenance is 7s. 9d. per head.

It is very pleasing to note that the Matron received a pension of £70 a-year. There are few things incidental to details of asylum management that deserve more prominent chronicling than such superannuation allowances. They lighten the burdens and cheer the path of all other asylum officials.

About 51 per cent. of the male patients are usefully employed, and among the women the percentage is higher. The Commissioners say they can report favourably "of the cleanliness, order and comfort of the wards," but they add that some of the wards are structurally very unsuitable for the proper treatment of the patients. We would ask why this is so, as it is known that large sums were quite lately spent in adding to and altering this asylum. We also learn from the Commissioners' Report that drinking water has to be carted daily from Shrewsbury. Possibly this may have been altered since the date of the report. The Commissioners say that the male attendants' mess-room is not well furnished, and Dr. Strange says he feels bound to differ with them about this. Who shall decide when doctors disagree?

### NEWCASTLE-UPON-TYNE ASYLUM.

This is the Pauper Asylum for the Borough of Newcastle, and it contains 352 patients. It would seem that the admissions were 104, the discharges 45, and the deaths 38. These numbers show that the percentage of recoveries was 27 9, and of the deaths 11·1. Of the admissions ten were suffering from general paralysis; eight were epileptics, and one-fourth were suffering from organic disease. These facts, as Dr. Callcott says, explain the low recovery rate.

